# A prospective, randomized, phase II study to assess the schemas of retreatment with Lutathera® in patients with new progression of an intestinal, well-differentiated neuroendocrine tumor (ReLUTH)

**DOI:** 10.1186/s12885-022-10443-4

**Published:** 2022-12-22

**Authors:** Emmanuel Deshayes, Eric Assenat, Laetitia Meignant, Manuel Bardiès, Lore Santoro, Sophie Gourgou

**Affiliations:** 1 Nuclear Medicine Department, Montpellier Cancer Institute (ICM), University of Montpellier, 208 Avenue des Apothicaires, 34298 Montpellier, France; 2 Institut de Recherche en Cancérologie de Montpellier (IRCM), INSERM U1194, University of Montpellier, Montpellier, France; 3 Medical Oncology Department, Montpellier Cancer Institute (ICM), University of Montpellier, Montpellier, France; 4 Medical Oncology Department, CHU Montpellier, University of Montpellier, Montpellier, France; 5 Clinical Research and Innovation Department, Montpellier Cancer Institute (ICM), University of Montpellier, Montpellier, France; 6 Biometrics Unit, Montpellier Cancer Institute (ICM), University of Montpellier, Montpellier, France

**Keywords:** 177Lu-DOTA-TATE, PRRT, Neuroendocrine tumor, Overall survival, Progression-free survival, Quality of life

## Abstract

**Background:**

Although neuroendocrine tumors (NET) are classed as rare, they have a high prevalence and their incidence is increasing. Effective treatment with lutetium ^17^-[177Lu]Lu-oxodotreotide (Lutathera®) is possible in patients with well-differentiated NET, improving progression-free survival (PFS), overall survival (OS), and quality of life (QoL). However, progression does occur. Retreatment with additional Lutathera® cycles is an option to extend PFS and OS. Two retreatment cycles are usually proposed. We aim to compare four versus two Lutathera® retreatment cycles in patients with new progression of a well-differentiated intestinal NET.

**Methods:**

This will be a multicenter, randomized, controlled, open-label, phase II study in France (ReLUTH). The aim is to evaluate the efficacy of retreatment with Lutathera® in patients with progressive intestinal NET (determined by somatostatin-receptor positive imaging) after previous treatment with two cycles of Lutathera®. Before randomization, all patients will have already received two Lutathera® retreatment cycles (7.4 GBq infusion each, 8 weeks apart). A total of 146 patients will be randomized (1:1) to two additional cycles of Lutathera® (7.4 GBq infusion each, separated by 8 weeks) or to no treatment (active surveillance). Primary objective: efficacy of two additional Lutathera® retreatment cycles compared to active surveillance over 6 months. Primary endpoint: disease control rate at 6 months from randomization (defined as Complete Response, Partial Response, and Stable Disease in the Response Evaluation Criteria In Solid Tumours) with an evaluation every 2 months. A secondary objective will be the safety, as well as the PFS, OS, and QoL. It is expected that the efficacy of retreatment will increase after two additional Lutathera® cycles, with no increased safety concerns.

**Discussion:**

Our prospective, randomized controlled study may lead to new recommendations for the use of Lutathera® in patients with intestinal progressive NET, and should confirm that four cycles will be more effective than two, with limited adverse impact on safety. Four Lutathera® treatment cycles have the potential to prolong life and improve quality of life in patients.

**Trial registration:**

ClinicalTrials.gov: NCT04954820.

**Supplementary Information:**

The online version contains supplementary material available at 10.1186/s12885-022-10443-4.

## Background

Neuroendocrine tumors (NET) arise from neuroendocrine cells in the endocrine and central nervous systems. They are classified as a rare disease with a low incidence, but are becoming increasingly common. In the USA, for example, the incidence per 100,000 people has risen from 1.09 to 1973 to 6.98 in 2012 [1]. Due to the slow-growing nature of NET, they have a high prevalence, affecting 35 out of 100,000 people [1]. Two-thirds of NET occur in the gastrointestinal tract [1].

In the NETTER-1 clinical trial [2], peptide receptor radionuclide therapy (PRRT) with Lutathera® ([^177^Lu]Lu-DOTA-TATE or [177Lu]Lu-oxodotréotide every 8 weeks (four doses) plus 30 mg octreotide LAR was compared with high-dose octreotide LAR (60 mg) every 4 weeks in patients with progressive and unresectable midgut, well-differentiated (grade [G]1, G2) NET with somatostatin-receptor positive imaging (SSTRi+). At follow-up (42 months), Lutathera had improved both the median progression-free survival (PFS) (28.4 vs. 8.5 months) and median overall survival (OS) (“not reached” vs. 27.4 months). The health-related quality of life in the NETTER-1 patients showed that Lutathera had a strong, positive impact on quality of life (global health status, hazard ratio [HR] 0.406), physical functioning (HR 0.518), role functioning (HR 0.580), fatigue (HR 0.621), pain (HR 0.566), diarrhea (HR 0.473), disease-related worries (HR 0.572), and body image (HR 0.425) [3]. Therefore, Lutathera treatment was approved by the European Medicines Agency and is now reimbursed in France for this specific indication, with four intravenous injections of 7.4 GBq of Lutathera® (delivered every 2 months. More recently [4], overall survival and long-term safety results have shown that Lutathera® did not significantly improve median overall survival versus high-dose, long-acting octreotide, but the 11.7-month difference may be considered clinically relevant. Long-term follow-up did not show any new toxicity concerns.

Despite these promising results, progression will occur within a variable time in most patients, leaving limited treatment options. Retreatment with additional cycles of Lutathera® may be a possibility. A few cohort studies have been published concerning retreatment. The most recent and largest one from Van der Zwan et al. [5] showed that in patients with gastroenteropancreatic (GEP)-NET (*n* = 168) and bronchial NET (*n* = 181), the median PFS was 14.6 months after retreatment with two additional cycles of  [177Lu]Lu-DOTA-TATE and the OS was significantly longer than in the non-randomized control group. Only patients with a PFS of ≥ 18.0 months from the first administration of initial PRRT were considered eligible for retreatment PRRT. The median PFS was 14.6, 14.7, and 20 months, respectively, for the foregut, midgut, and hindgut, while the OS was 33.9, 23.1, and 56.9 months. Interestingly, safety was similar between the salvage and initial PRRT groups: no grade 3/4 renal toxicity occurred, and hematological toxicities (acute myeloid leukemia and myelodysplastic syndrome) were similar to the group of patients who received the initial treatment (four cycles). Smaller cohort studies [6–10] showed that retreatment with [177Lu]Lu-DOTA-TATE led to an improvement in survival with an acceptable safety profile (in particular, there was no increase in the incidence of renal or hematotoxicity compared to “first PRRT”), even in patients who received eight or more cycles of  [177Lu]Lu-DOTA-TATE.

Clinical practice regarding the number of new cycles is heterogeneous and most teams perform only two additional cycles (every 8 weeks), which were recently showed to be safe and effective [5]. The few studies to evaluate retreatment have been cohort studies. There is a need for a prospective trial to assess the effectiveness, safety, and quality of life after four cycles of retreatment with Lutathera® in patients with intestinal progressive SSTRi + NET, with the objective of a higher disease control rate. Accordingly, we plan to study the impact of two additional cycles compared to a controlled arm in active surveillance in the ReLUTH (retreatment with Lutathera®) trial. Different stratification factors will be used in the study, as follows: (a) the time of disease control after the “first” PRRT (< 18 months vs. ≥ 18 months) – patients with a history of a durable PFS after initial PRRT tend to have long-lasting PFS after salvage treatment (*p* = 0.04) [9]; (b) type of progression (radiologic vs. no radiologic); (c) renal function (glomerular filtration rate (GFR) < 60 mL/min vs. ≥ 60 mL/min), as it may have an impact on disease control [9] and OS [6]; (d) intercurrent line of treatment between “first” and “second” PRRT, as it is currently unclear (Yes vs. No).

The results of our prospective, randomized, controlled study may lead to new recommendations for the use of PRRT in patients with intestinal progressive SSTRi + NET. ReLUTH would provide strong statistical data to confirm that in patients retreated for intestinal progressive NET, two additional cycles of Lutathera® will be safe and effective compared to only two cycles and will have a beneficial impact on quality of life. If our hypothesis is confirmed, the results of our study would be able to prolong life and improve quality of life in patients.

## Methods

### Study design and objectives

**Fig. 1:**
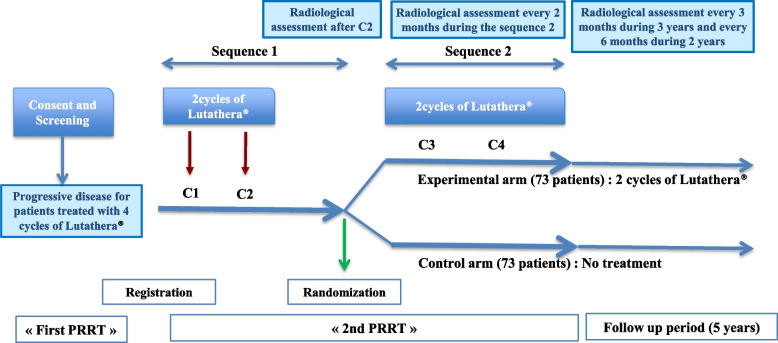
Study flow chart

ReLUTH will be a prospective, multicenter, randomized, controlled, open-label, phase II trial performed throughout France (Fig. 1 and Additional file 1). The primary objective will be to evaluate the efficacy of two additional cycles of Lutathera® (one injection every two months) compared to active surveillance during 6 months in patients who have already been retreated with two cycles. The primary endpoint will be the disease control rate (DCR) at 6 months from randomization (defined as Complete Response, Partial Response, and Stable Disease in the Response Evaluation Criteria In Solid Tumours (RECIST) v1.1) with an evaluation every 2 months.

As secondary objectives, we intend to evaluate the safety (using National Cancer Institute-Common Terminology Criteria (NCI-CTCAE) v5.0), rPFS (defined as the time from randomization until documented disease progression on radiological tumor assessment (as evaluated by an independent central review by radiologists blinded to the treatment assignments according to RECIST v1.1) or death from any cause, whichever occurs first) and PFS (defined as the time from randomization until documented disease progression on radiological tumor assessment, as evaluated in an independent central review by radiologists blinded to the treatment assignments according to RECIST v1.1), and OS (defined as the time from randomization until death from any cause). Quality of life (QoL) will also be assessed during and after treatment in both arms (assessed using the European Organization for Research and Treatment of Cancer (EORTC) QLQ-C30 and GI.NET21 questionnaires).

The expected benefit will be an increase in the efficacy of retreatment after two additional cycles of Lutathera® compared to active surveillance in patients with an intestinal NET in relapse. The risk-benefit balance of the study will be continuously evaluated by the ICM Clinical Research Pharmacovigilance Unit and discussed in the periodic safety reports.

The study was approved by a national Ethics Committee and will be performed according to Good Clinical Practice and the Declaration of Helsinki. The trial was prospectively registered on ClinicalTrials.gov: NCT04954820. The study protocol adheres to SPIRIT guidelines.

### Patients

Recruitment began in October 2021 and will be ongoing for 3 years. The intention is to recruit 176 patients with an intestinal NET previously treated by four cycles of Lutathera® who are presenting progression (clinic, biologic, and/or radiologic) and whose retreatment with Lutathera® has been decided by a multidisciplinary tumor board (RENATEN). This number will allow 146 patients to be randomized. All patients will be followed for 60 months.

The principal inclusion criteria will be as follows: age ≥ 18 years; histologically-proven intestinal G1 or G2 NET; previous treatment with four cycles of Lutathera® (defined as “First PRRT”) and disease control after “First PRRT” ≥ 12 months; presentation of disease progression (clinic, biologic, and/or radiologic) after the first PRRT; decision to retreat with Lutathera® (defined as “Second PRRT”) validated by RENATEN and/or a multidisciplinary tumor board and in the scope of the French reimbursement process; Eastern Cooperative Oncology Group (ECOG) performance status 0–2; life expectancy ≥ 6 months, as prognosticated by the physician; SSTRi + disease within 4 months prior to randomization (may be positron emission tomography imaging [^68^Ga-based SSTR analogues] or scintigraphy imaging [^111^In-pentetreotide or ^99m^Tc-octreotide]; ≥ 90% of lesions must be SSTRi + with a significant uptake [beyond liver or surrounding tissue], measurable disease per RECIST 1.1 (Additional file 1); on CT/MRI scans, defined as ≥ 1 lesion with ≥ 1 cm in longest diameter, and ≥ 2 radiological tumor lesions in total; adequate bone marrow reserve [Hb > 8 g/dl, neutrophils ≥ 1500/mm³, and platelets ≥ 80 000/mm³]). All patients will be required to sign an informed consent before inclusion.

Principal exclusion criteria will include: no response (i.e., no complete response [CR], partial response [PR], or stable disease [SD]) to “first PRRT”; radiological progression after two cycles of “Second PRRT” according to RECIST version 1.1; G4 hematotoxicity and/or nephrotoxicity during the initial PRRT, or unresolved adverse events categorized as G2 or higher (as per the CTCAE v5.0 from previous PRRT cycles or any other therapy for NET, excluding alopecia and peripheral neuropathy); pancreatic NET; neuroendocrine carcinoma; patients with prior external beam radiation therapy to > 25% of the bone marrow; severe renal impairment (GFR according to the Modification of Diet in Renal Disease < 40 mL/min or nephrotic syndrome) or hepatic insufficiency (alanine aminotransferase/aspartate aminotransferase > 2.5 x the upper limit of normal [ULN], or > 5 x ULN if liver function abnormalities are due to the underlying malignancy, and/or total serum bilirubin > 2.5 x ULN); any other uncontrolled concomitant disease or brain metastases (unless they have been treated and stabilized for at least 24 weeks prior to enrolment in the study; patients with a history of brain metastases must have had a head CT scan with contrast or MRI to document stable disease prior to enrolment in the study); history of another solid tumor in the 5 years before inclusion, apart from treated and controlled cancer in the cervix and skin cancer (basal or squamous cell).

### Treatments

Before randomization, all patients will have received two retreatment cycles of Lutathera® (7.4 GBq infusion each, separated by 8 weeks) (Fig. 1). A radiological tumor assessment will be performed after two cycles. Patients with progression during the first sequence of Lutathera® retreatment will be considered as screen failure and will be followed until the end of the study.

### Randomization and masking

Randomization (1:1) will be performed using EnnovClinical software. Patients will be randomized to the experimental arm, consisting of two additional cycles of Lutathera® (7.4 GBq infusion each, separated by 8 weeks), or the control arm with no treatment (active surveillance) (Fig. 1).

### Statistical considerations

A median PFS of 15 months [11] after two cycles of Lutathera® for retreatment corresponds to around a 65% disease control rate 6 and 10 months from randomization and inclusion, respectively. To detect a minimal difference of 20% in disease control rate at 6 months post-randomization (65% vs. 85%) between the control and experimental groups, respectively, with a power of 80% and a 5% two-sided significance level, a total of 146 patients is required for randomization (73 patients per arm). Non-evaluable patients will be considered as failure. We estimate that around 176 patients will need to be included to ensure that 146 patients are randomized.

All analyses will be performed on an intention-to-treat (ITT) basis, defined as all randomized patients, while the safety population will comprise all treated patients who received at least one dose of treatment after inclusion. The primary endpoint will also be analyzed in the per-protocol (PP) population, defined as all eligible patients (patients with no major violations of the inclusion/non-inclusion criteria) and evaluable patients (randomized treated patients with a minimum of two evaluations).

Categorical variables will be reported with numbers and frequencies and continuous variables with medians and ranges. Qualitative variables will be compared using chi-square or Fisher’s exact tests, while quantitative variables will be compared using the Kruskal-Wallis test. The DCR will be reported using percentages and 95% confidence intervals (CI) (binomial exact method). Event-free survival (PFS, OS) will be estimated using the Kaplan-Meier method and compared using the stratified log-rank test. Statistical analyses will be performed using STATA 16.0 (StatCorp, College Station, TX, USA) and SAS 9.4 software.

### Ancillary study

There is a growing interest in dosimetry, to document the irradiation delivered and potentially allow treatment personalization [12]. Indeed, the amount of radioactivity injected into patients is fixed (7.4 GBq), but the dose deposit to organs and tumors may present with large variations related to different pharmacokinetics between patients. Two studies have either been completed or are ongoing [13, 14] and preliminary results are promising [11]. Performing clinical dosimetry requires acquiring imaging data on dose limiting organs (especially the kidney and bone marrow) and tumors in a salvage PRRT setting. This ancillary study will collect data from around 50 patients in centers that already perform dosimetry for Lutathera®. Correlation with clinical outcomes (disease control rate and toxicity) will be studied. This will allow assessment of the absorbed dose-effect relationship in patients treated with up to eight Lutathera® cycles.

### Availability of data and materials

The database will be hosted by the Institut du Cancer Montpellier, Montpellier, France. Participant data will be available upon reasonable request and with the completion of a contract between the sponsor and the applicant.

### Role of the funding sources

The study was funded by the Direction Generale de l’Offre de Soins (reference no. PHRC-K 20–034) after international review by an expert board. The funder had no role in study design, collection, analysis or interpretation of data, writing of the report or decision to submit the paper for publication.

## Discussion

No prospective trial has evaluated retreatment with Lutathera®. Our prospective, randomized controlled study may lead to new recommendations for the reuse of Lutathera® in patients with intestinal progressive NET. Four additional cycles should be more effective than two in terms of survival and quality of life, with constant monitoring of toxicities.

One of the challenges of this study will be to obtain a sufficient number of randomized patients to receive the expected number of cycles. Indeed, withdrawal of patients due to toxicities or disease progression during the first two cycles of treatment is a distinct possibility. Another challenge is the large number of participating centers (*n*=22), many of which opened within the 6 first months of the study. However, this number will allow us to include the required number of patients.

The ancillary dosimetric study will be of great importance. Although treatment will be based on fixed infusions (7.4 GBq) in every cycle, there will be a wide variability in dose deposit to tumors and organs in each patient. The dose-effect relationship is not obvious and more data has to be collected to better understand how the dose to tumors affects response, and how the dose to healthy organs may cause toxicities. Coordinating a multicentric ancillary dosimetry study will be difficult, however. Issues may arise concerning, for example, preliminary assessment of the qualification of each associated center, standardization of acquisition and reconstruction procedures (performed locally by each participating center), definition of quality assurance and quality control adapted to the purpose, image transfer in a format that allows centralized dosimetry, and ensuring that the anonymization process (performed locally by each participating center) does not prevent performance of the dosimetry study [15]. Communication between medical physicists in each center was a prerequisite for the successful implementation of this ancillary study. Image acquisition and dosimetry protocols have been standardized as much as possible, taking into account the specificities of each nuclear medicine department.

## Supplementary Information

**Additional file 1.** Schedule of enrolment, interventions, and assessments.

## Data Availability

Not applicable.

## Abbreviations

CI: confidence interval
CR: complete response
DCR: disease control rate
ECOG: Eastern Cooperative Oncology Group
EORTC: European Organization for Research and Treatment of Cancer
G: grade
GEP: gastroenteropancreatic
GFR: glomerular filtration rate
HR: hazard ratio
ITT: intention-to-treat
NCI-CTCAE: National Cancer Institute-Common Terminology Criteria for Adverse Events
NCI-CTC: National Cancer Institute-Common Terminology Criteria
ORR: overall response rate
OS: overall survival
PFS: progression-free survival
PP: per protocol
PR: partial response
PRRT: peptide receptor radionuclide therapy
RECIST: Response Evaluation Criteria In Solid Tumours
RT: radiotherapy
SD: stable disease
SSTRi+: somatostatin-receptor positive imaging
ULN: upper limit of normal
