# Supplementary material for: A prospective, randomized, phase II study to assess the schemas of retreatment with Lutathera® in patients with new progression of an intestinal, well-differentiated neuroendocrine tumor (ReLUTH)
Source: BMC Cancer. 2022 Dec 22;22:1346. doi: 10.1186/s12885-022-10443-4 (PMC9773621; doi:10.1186/s12885-022-10443-4)
Supplement: Supplementary file 1 — Additional file 1. Schedule of enrolment, interventions, and assessments. [file 12885_2022_10443_MOESM1_ESM.docx]

**Additional File 1.** Schedule of Enrolment, Interventions, and Assessments.

|  | Baseline | | | | **INCLUSION** | Treatment period of Sequence 1 | | | | Evaluation pre-R | **RANDOMIZATION** | Treatment period of Sequence 2 | | | | Evaluations at 4M & 6M post-R | Follow-up period |
| --- | --- | --- | --- | --- | --- | --- | --- | --- | --- | --- | --- | --- | --- | --- | --- | --- | --- |
|  |  |  |  |  |  | Cycle 1  D1-D56 | | Cycle 2  D57-D112 | |  |  | Cycle 3  D113-D168 | | Cycle 4  D169-D224  2M post-R | |  |  |
|  | M -4 | D  -30 | D  -7 | D  -4 |  | D1 | D3 & D8 | D57 (C2D1) | D59 and D64 (C2D3 & C2D8) | D113 |  | D113 (C3D1) | D115 & D120 (C3D3 & C3D8) | D169 (C4D1) | D171 & D176 (C4D3 & C4D8) | D225 & D281 | Every 3M over 3Y, then every 6M over 2Y |
| Informed consent |  | X |  |  |  |  |  |  |  |  |  |  |  |  |  |  |  |
| Review of eligibility criteria |  |  |  | X |  |  |  |  |  |  |  |  |  |  |  |  |  |
| Complete medical history |  | X |  |  |  |  |  |  |  |  |  |  |  |  |  |  |  |
| Adverse event |  | X |  |  |  | X |  | X |  |  |  | X |  | X |  | X | X |
| Concomitant medications |  | X |  |  |  | X |  | X |  |  |  | X |  | X |  | X | X |
| Menopausal status |  | X |  |  |  |  |  |  |  |  |  |  |  |  |  |  |  |
| Pregnancy test (if applicable) |  |  |  | X |  |  |  |  |  |  |  |  |  |  |  |  |  |
| Clinical examinations (ECOG, height, weight, blood pressure, pulse) |  |  | X |  |  | X |  | X |  |  |  | X |  | X |  | X | X |
| Hematology* & blood chemistry** |  |  | X |  |  | X *^a^* |  | X *^a^* |  |  |  | X *^a^* |  | X *^a^* |  | X *^a^* | X *^b^* |
| Chomogranin A and 5HIAA |  |  | X |  |  |  |  |  |  | X |  |  |  |  |  | X | X *^c^* |
| SRI*** | X |  |  |  |  |  |  |  |  |  |  |  |  |  |  |  | X *^d^* |
| CT scan and/or MRI**** |  | X |  |  |  |  |  |  |  | X |  |  |  | X |  | X | X |
| PET scan (optional) |  | X |  |  |  |  |  |  |  |  |  |  |  |  |  |  |  |
| Head CT scan and/or MRI (only if brain metastasis present) |  | X |  |  |  |  |  |  |  | X |  |  |  | X |  | X | X |
| SPECT/CT & whole-body planar image (only for patients in ancillary study) |  |  |  |  |  | X *^e^* | X *^e^* | X *^e^* | X *^e^* |  |  | X *^e^* | X *^e^* | X *^e^* | X *^e^* |  |  |
| Blood sample (only for patients in ancillary study |  |  |  |  |  | X *^e^* | X *^e^* | X *^e^* | X *^e^* |  |  | X *^e^* | X *^e^* | X *^e^* | X *^e^* |  |  |
| EORTC QLQ C-30 & GI.NET21 questionnaires |  | X |  |  |  | X |  | X |  |  |  | X |  | X |  | X | X *^f^* |
| Cycle of Lutathera® |  |  |  |  |  | X |  | X |  |  |  | X *^g^* |  | X *^g^* |  |  |  |
| Vital status |  |  |  |  |  |  |  |  |  |  |  |  |  |  |  |  | X |

5HIAA, 5-hydroxyindoleacetic acid; C, cycle; CT, computed tomography; D, day; EORTC European Organization for Research and Treatment of Cancer; M, months; PET, positron emission tomography; R, randomization; SPECT, single-photon emission computed tomography; SRI, Somatostatin receptor imaging; Y, years.

* Hematology with red blood cell count, hemoglobin, hematocrit, platelet count and white blood cell count (which must include differential neutrophil, lymphocyte, monocyte, basophil and eosinophil counts). ** Blood chemistry: aspartate amino transferase, alanine amino transferase, total bilirubin, alkaline phosphatase, calcium, creatinine, serum creatinine clearance calculated according to the Cockcroft Gault method, lactate dehydrogenase, glucose, urea, albumin, prothrombin time. *** SPECT agent: Octreotide® or Octreoscan®, PET imaging Gallium-based somatostatin analog. ****: CT scan of the abdomen, pelvis and chest or hepatic MRI and CT scan of the pelvis and chest without injection; RECIST criteria version 1.1.

*^a^* For all patients, hematology and blood chemistry should be realized every month during Sequence 1, Sequence 2 and throughout the follow-up period (within 5 days of visit date). *^b^* For all patients, hematology and blood chemistry should be realized every 3 months for 3 years and then every 6 months for 2 years (within 5 days of visit date). *^c^*chomogranin A and 5HIAA should be done every 6 months during the follow-up period. *^d^* SRI should be realized at the follow-up M12 post-randomization (follow-up number 2). *^e^* SPECT/CT and whole-body planar image should be done at 4±2 h, 24±6 h, 72±6 h, and 192±6 h; blood samples (2 ml) will be drawn at 0.5, 1.0, 2.5, 4, 8, and 24 h after the start of Lutathera administration; additional samples will be collected at 72±6 h and 192±6 h after injection (synchronized with imaging sessions). *^f^* Questionnaires will be assessed every 3 months over 1 year, then every year over 4 years. *^g^* Only in the experimental arm.
